# Perinatal Outcomes in Extra vs. Transperitoneal Cesarean Delivery: A Systematic Review and Meta-Analysis of Randomized Controlled Trials

**DOI:** 10.3390/jcm15010191

**Published:** 2025-12-26

**Authors:** Manal Massalha, Kamel Mattar, Rula Iskander, Mais Abu Nofal, Ido Izhaki, Raed Salim

**Affiliations:** 1 Department of Obstetrics and Gynecology, Emek Medical Center, Afula 1834111, Israel; manal.massalha@clalit.org.il (M.M.); rola_is@clalit.org.il (R.I.); 2 The Ruth and Bruce Rappaport Faculty of Medicine, Technion-Israel Institute of Technology, Haifa 3200003, Israel; 3 Department of Obstetrics and Gynecology, Holy Family Hospital, Nazareth 1641105, Israel; kamel.mattar@mail.huji.ac.il (K.M.); maisabunofal@gmail.com (M.A.N.); 4 Department of Evolutionary and Environmental Biology, University of Haifa, Haifa 3103301, Israel; izhakido@gmail.com; 5 Azrieli Faculty of Medicine, Bar-Ilan University, Safed 1311502, Israel

**Keywords:** extraperitoneal cesarean delivery, maternal outcome, meta-analysis, neonatal outcome, randomized controlled trials

## Abstract

**Background:** Despite the advantages of extraperitoneal cesarean delivery (EPCD) indicated by observational studies, there is little accurate evidence supporting this technique, and the studies performed have included small numbers of participants. We aimed to examine intra- and postoperative maternal and neonatal outcomes in EPCD compared with transperitoneal CD (TPCD). **Methods:** Six databases restricted to English-language studies were searched from inception to August 2025. Only peer-reviewed randomized controlled trials (RCTs) directly comparing EPCD and TPCD were included. Study quality was evaluated using the Cochrane Risk of Bias tool. Primary neonatal and primary maternal outcomes were the Apgar score and postoperative pain, respectively. The protocol was prospectively registered in PROSPERO (#CRD42023420365). **Results:** Of the 69 reports identified, seven RCTs comprising 758 women (379 per group) were eligible. Data for 1 min Apgar scores were insufficient for analysis because standard deviations were missing for most studies. Five-minute Apgar scores were comparable between the two techniques (*p* = 0.91). Incidence of umbilical artery pH < 7.2 was higher in the EPCD group than in the TPCD group (7.9% vs. 2.3%, respectively; *p* = 0.047). Mean incision-to-delivery time was longer in the EPCD group (7.5 ± 5.0 min) compared with the TPCD group (6.2 ± 3.7 min, *p* = 0.017). Postoperative pain at 24 h was lower after EPCD (*p* < 0.001), and time to first gas passage was shorter (7.4 ± 2.7 h vs. 14.7 ± 2.7 h, *p* < 0.001) compared with TPCD. Other perioperative outcomes were comparable. **Conclusions:** The safety of EPCD for the neonate requires further investigation. Maternal postoperative pain and time to gas passage were favorable in EPCD.

## 1. Introduction

Cesarean delivery (CD) is the most frequent surgical operation performed worldwide [1]. Though the rates of maternal morbidity and mortality related to the procedure are relatively low, it is vital that surgeons consistently use evidence-based techniques that have been shown to reduce surgical complications. Several aspects of the surgery are supported by evidence with a high level of certainty. Nevertheless, there is little available evidence regarding several other aspects, and their practice is largely based on the surgeon’s preference [1].

The standard technique for CD in most medical institutions around the world includes opening the parietal peritoneum, i.e., transperitoneal CD (TPCD), and delivering the infant through a transverse incision in the lower uterine segment. TPCD inevitably leads to exposure of the peritoneal cavity to blood, amniotic fluid, vernix, and mechanical irritation that may potentially generate pain, nausea and vomiting, intestinal paralysis, future adhesions, and infertility [2,3,4,5]. Before the widespread use of antibiotics, it was speculated that an extraperitoneal approach to CD may protect intraperitoneal organs from intrauterine infection, thus avoiding potential harmful effects and reducing morbidity [6,7,8,9]. Additionally, observational studies on extraperitoneal CD (EPCD) indicated that this approach leads to rapid postoperative recovery and a reduced need for postpartum analgesic use [10,11,12,13,14]. In original reports, EPCD is initiated with a Pfannenstiel incision, followed by blunt midline separation of the rectus muscles to access the preperitoneal space. The preperitoneal fat is swept aside to identify the medial umbilical fold and bladder landmarks, allowing for medial and inferior retraction of the bladder and exposure of the lower uterine segment without opening the peritoneum. The lower uterine segment is then incised and digitally extended bilaterally to enable extraperitoneal fetal delivery [10,11,12,13,14,15].

However, despite the theoretical advantages of the extraperitoneal technique, there is little evidence supporting its practice, and the relevant studies performed included only relatively small numbers of participants [14,15,16]. Additionally, data regarding neonatal outcomes in terms of immediate postpartum condition and events related to extraction are inconsistent and occasionally not reported. Accordingly, the present meta-analysis aimed to combine the available level 1 evidence and compare EPCD with TPCD, with respect to neonatal outcome and maternal complications. We focused only on level 1 evidence; thus, we included only randomized controlled trials (RCTs) that compared the two techniques.

## 2. Materials and Methods

This systematic review and meta-analysis were performed according to the principles of the Preferred Reporting Items for Systematic Reviews and Meta-analysis [17] (Supplementary Checklist S1). The study protocol was registered with PROSPERO (Preferred Reporting Items for Systematic Reviews and Meta-Analyses) on 21 April 2023 (registration number: CRD42022311879).

### 2.1. Search Strategy

MEDLINE, EMBASE, PubMed, Ovid MEDLINE, ClinicalTrials.gov, and the Cochrane Library were searched from inception to April 2023 with language restriction to English only. Later, we extended the range until August 2025 because additional eligible articles suitable for inclusion were published. Search terms were as follows: (extraperitoneal OR French) AND (transperitoneal OR Intraperitoneal OR Misgav–Ladach) AND (Cesarean OR Caesarean OR Cesarean Section [MH]). Only peer-reviewed RCTs that compared EPCD with TPCD were included. Three authors (M.M., K.M., and R.S.) independently performed the search, all appropriate articles were retrieved, and the references of the relevant articles were manually reviewed to identify additional studies.

### 2.2. Selection, Eligibility, and Quality of the Studies

All randomized, peer-reviewed trials that assessed intra- and postoperative maternal and neonatal outcomes for EPCD compared with TPCD were included in this meta-analysis. Abstract-only and editorial publications were excluded. Disagreement concerning the trials suggested to be included was resolved through discussion and the establishment of consensus between the three authors.

### 2.3. Data Extraction

The data were extracted from the text, graphs, and tables of each of the selected articles on three distinct occasions to validate the precision of the data collected. We developed descriptive tables for the study and population characteristics of the eligible publications. The tables included the first author, publication year, country, and study design. In addition, sample size, maternal age, gestational age at delivery, and primary outcome were also included in the baseline descriptive data of the enrolled trials.

### 2.4. Outcome Measures

The primary neonatal outcome was the Apgar score in the EPCD group compared with the TPCD group. This outcome was selected since prolonged extraction or the need for assisted mechanical extraction of the fetus, which has been reported in this type of operation, may affect the Apgar score. Secondary neonatal outcomes included incision to delivery time, umbilical cord artery pH, and neonatal trauma related to the extraction technique. The primary maternal outcome was postoperative pain assessed within the first 24 h by the Visual Analogue Scale (VAS). Secondary maternal outcomes were intraoperative nausea/vomiting, operative time, time to gas passage, perioperative change in hemoglobin level, and maternal length of stay.

### 2.5. Risk of Bias Assessment

The Cochrane Risk of Bias tool for RCTs was utilized [18], and the quality of the included studies was assessed accordingly by three independent authors (M.M., R.I., and R.S.). Various aspects of bias were evaluated, including the randomization process, deviation from the intended interventions, missing outcome data, measurement of the outcome, and selection of the reported results. The overall risk of bias was judged to be low if all examined aspects were considered as such, and high if at least one domain was considered to be as such or the study was judged to present some concerns in multiple domains.

### 2.6. Data Analysis

We examined intraoperative and postoperative maternal and neonatal outcomes comparing EPCD with TPCD. Quantitative results for continuous outcomes are presented as means with standard deviations (SDs), and pooled effects were calculated using the weighted mean difference (WMD). For outcomes reported as medians with ranges (minimum and maximum), means and SDs were estimated. As all studies included in the present analyses had sample sizes ≥ 25, we estimated the SD as range/4 for sample sizes between 15 and 70, and as range/6 when the sample size was ≥70, in accordance with the recommendations of Hozo et al. [19] In addition, for sample sizes ≥ 25, the median itself was used as an estimator of the mean [19]. Meta-analysis was performed using a random-effects model based on the DerSimonian and Laird method.

Categorical outcomes were measured using relative risk (RR). Pooled differences in means and pooled relative risks were calculated by combining the results of all included studies while accounting for the appropriate statistical weight of each study. Statistical analyses and graphical data visualization were performed using open-source software OpenMeta (version 10.12) [Analyst].

Statistical heterogeneity was assessed by Cochrane’s Q test of heterogeneity (*p* < 0.1 was considered statistically significant), and inconsistency was evaluated by the I^2^ statistic (value > 50% was judged to suggest significant heterogeneity between trials). Sensitivity analyses were performed to assess the robustness of the pooled estimates for the maternal and neonatal outcomes that differed significantly between groups. To evaluate the influence of individual trials on the pooled effect estimates and heterogeneity, we conducted sequential exclusion of individual trials and compared fixed- and random-effects models. Sensitivity analyses were not performed where fewer than three trials underwent meta-analysis.

## 3. Results

### 3.1. Study Selection

**Figure 1:**
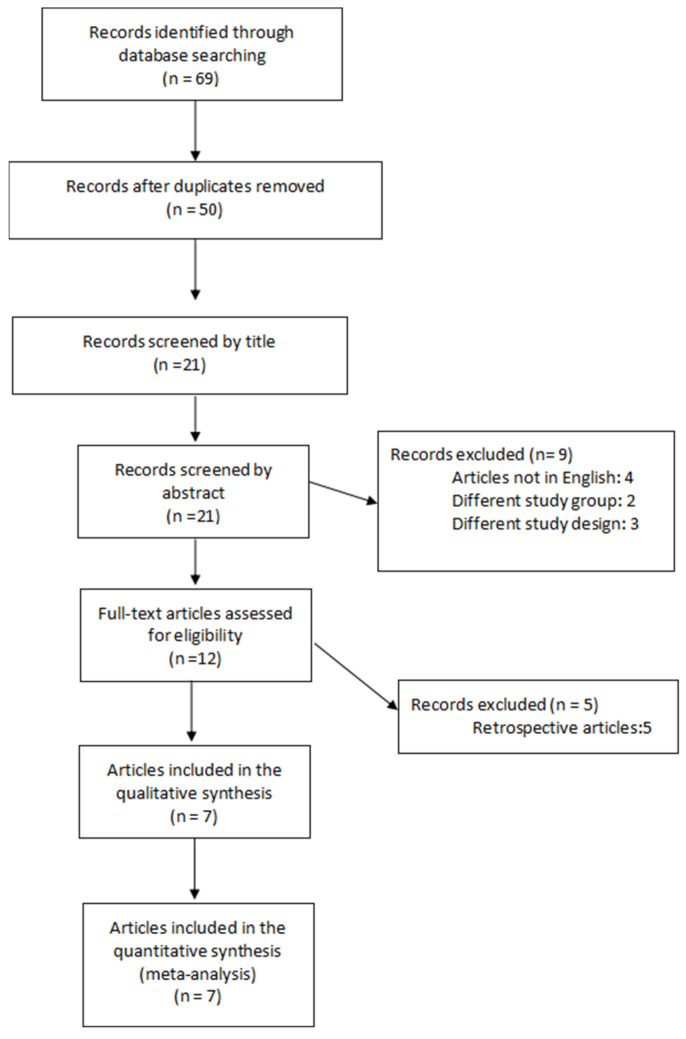
PRISMA flowchart outlining study selection.

Following the search carried out according to the criteria defined in the Methods section, 69 articles were identified, of which seven were RCTs and considered eligible and included in the meta-analysis [20,21,22,23,24,25,26]. The PRISMA flowchart of the study selection is presented in Figure 1. The seven eligible RCTs included a total of 758 women (379 in the EPCD group and 379 in the TPCD group) who underwent either primary or repeat CD. Four of the seven trials were registered in the clinical trials registry [20,21,24,25], and the enrollment of the first participant was prior to the registration date, except for one [20].

The CD anesthesia protocol was similar between the groups in all trials except that of Dimassi et al. [20], where intrathecal morphine was added to the TPCD group only. Postoperative analgesic administration was reported in five trials [20,21,23,24,25]. All five reported a similar protocol between the groups. In four trials, the extraperitoneal approach was accomplished by separating the paired rectus muscles in the midline, and the fascia was then incised transversely [21,22,23,24], while in three, the rectus muscles were separated on one side only, lateral to the linea alba [20,25,26].

### 3.2. Risk of Bias

One trial was deemed to have a low risk of bias, and six trials were considered to be at high risk of bias (Supplementary Figure S1).

### 3.3. Study Characteristics

The baseline descriptive data of the enrolled trials in the meta-analysis are shown in Supplementary Table S1. The incidence of accidental peritoneal opening in the EPCD groups ranged from 0% to 50%. Basic demographic and obstetric variables of the women included in the meta-analysis in both groups are presented in Table 1. Mean maternal age was 30.1 ± 3.5 years among the EPCD group and 30.9 ± 3.0 years in the TPCD group (*p* < 0.001). The rates of nulliparous women were 53.7% and 45.9% in the EPCD and TPCD groups, respectively (*p* < 0.001). The history of one CD or more was significantly higher in the TPCD group compared with the EPCD group (46.7% and 36.8%, respectively; *p* < 0.001).

### 3.4. Synthesis of Results

**Figure 2:**
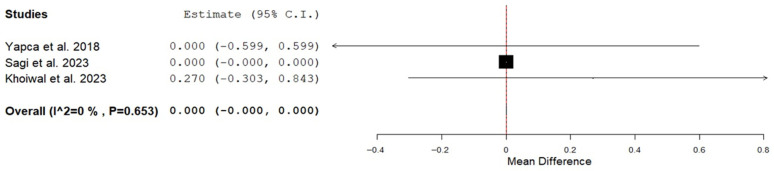
Forest plot of Apgar score at 5 min in the extra-compared to transperitoneal cesarean delivery [24,25,26].

Incision to delivery time was 7.5 ± 5.0 min in the EPCD compared with 6.2 ± 3.7 min in the TPCD (weighted mean difference (WMD): 2.23; 95% CI: 0.40 to 4.06; *p* = 0.017; I^2^ = 98.0%) (Table 2). Sensitivity analysis indicated that the overall pooled mean difference for incision-to-delivery time was not materially affected by the exclusion of any individual trial, with pooled estimates ranging from 1.5 to 2.9. A meta-analysis of Apgar scores at 1 min could not be conducted, as six of the seven studies lacked the necessary data to compute standard deviations. Apgar scores at 1 and 5 min, as reported in each article, did not differ significantly between the EPCD and the TPCD groups within each trial. We performed a meta-analysis on the Apgar score at 5 min, and the score was similar between the groups (Figure 2 and Table 2). Mean umbilical artery pH was 7.302 ± 0.02 and 7.300 ± 0.00 in the EPCD and the TPCD groups, respectively (WMD: 0.01; 95% CI: 0.004 to 0.016; *p* = 0.001; I^2^ = 0%). The rate of umbilical cord artery pH < 7.2 was 7.9% and 2.3% in the EPCD and TPCD, respectively (*p* = 0.047) (Table 2). Umbilical cord artery pH < 7.2 was reported in 2 studies (336 neonates) [24,25]. In one study, the authors summarized that all neonates had an uneventful outcome [24], and in the second, the authors stated that none had a pH < 7.0 [25]. The necessity of Teissier spatula use to assist deliveries in cases of limited space for fetal extraction and its consequences was not persistently reported for the EPCD group and thus was not analyzed. Additionally, rates of artificial ventilation, neonatal intensive care unit admission, and any neonatal trauma were also not reported and thus not analyzed.

**Figure 3:**
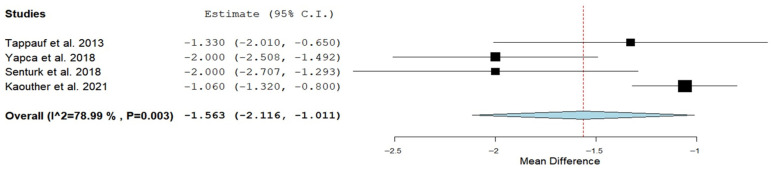
Forest plot of postoperative pain assessed by Visual Analogue Scale score at 24 h in the extra-compared to transperitoneal cesarean delivery [20,21,23,24].

Intraoperative outcomes were comparable (Table 3). Postoperative pain assessed by Visual Analogue Scale (VAS) score at 24 h was 2.7 ± 0.9 and 4.4 ± 1.2 in the EPCD group compared with the TPCD group (WMD: −1.56; 95% CI: −2.12 to −1.01; *p* < 0.001; I^2^ = 78.99%) (Figure 3 and Table 3). Sensitivity analysis of mean postoperative pain at 24 h indicated that the overall pooled effect size was robust, as exclusion of any single trial did not materially alter the pooled mean difference (range: 1.39–1.81). VAS score was also lower in the EPCD group compared with the TPCD group at 12 and 18 h (*p* = 0.039 and 0.001, respectively). At 6 h, the VAS score was also lower, but the difference was not significant (*p* = 0.079). Time to maternal gas passage was 7.4 ± 2.7 h in the EPCD group compared with 14.7 ± 2.7 h in the TPCD group (WMD: −4.64; 95% CI: −5.53 to −3.76, *p* < 0.001; I^2^ = 33.03%). Sensitivity analysis of the mean time to first gas passage indicated that the overall pooled effect size was robust, as exclusion of any single trial did not materially alter the pooled mean difference (range: 4.5–5.1). Length of hospital stay was similar between the groups (*p* = 0.49) (Table 3).

## 4. Discussion

### 4.1. Main Findings

The present review and meta-analysis included seven RCTs that compared EPCD and TPCD with regard to neonatal and maternal outcomes. The results showed that the time from incision to delivery was longer for EPCD by ~2 min compared with TPCD. It was not possible to analyze the impact of this delay on the Apgar score at 1 min due to an inability to calculate the standard deviations. Apgar scores at 5 min were the same, regardless of whether the peritoneum was opened or remained intact. Mean umbilical artery pH was almost similar between the groups, though it differed statistically. This difference does not necessarily imply clinical relevance. Nevertheless, the rate of umbilical cord artery pH < 7.2 was higher among neonates born by EPCD compared with TPCD. The need to use Teissier spatulas to assist deliveries in cases of limited space and its neonatal consequences were not persistently reported for the EPCD group and thus were not analyzed. Pain assessed every 6 h during the first 24 h after surgery and the time until gas passage were all lower for EPCD compared with TPCD. Additional intra- and postoperative outcomes examined were similar. The significant differences in maternal and neonatal outcomes found between the groups persisted following sensitivity analyses.

### 4.2. Comparison with Existing Literature

CD is the most common abdominal operation in the world, and the rate of planned CDs is increasing in many countries [1]. Accordingly, the use of surgical techniques based on high-level evidence is required. The development of extraperitoneal surgical techniques emerged at a time when infection was a major concern. The concept of returning to the extraperitoneal technique may be challenged in an era where antibiotics are readily accessible, and the infectious complication rate has dropped significantly. Based on the publications cited in this article, the extraperitoneal approach is currently practiced in a relatively small number of centers, mainly in Europe and parts of Asia [20,21,22,23,24,25,26], whereas the transperitoneal approach remains the dominant global standard. The extraperitoneal technique could present several additional short-term benefits. Based on the results of the current study, EPCD is associated with a significant reduction in maternal postoperative pain over the entire first 24 h. This outcome was persistently reported among trials analyzed. Lower levels of postoperative pain are associated with improved patient comfort, a reduced rate of pulmonary function disorders, and earlier mobilization, which collectively may facilitate early discharge [21]. Furthermore, earlier return of bowel function was also observed among women who had EPCD. Both postoperative pain reduction and early return of bowel function may be related to the fact that the peritoneum was not entered and, as a result, no irritation to the intestine or diaphragm by blood or amniotic fluid occurred [20,21,22,23,24].

Nevertheless, several issues still need to be explored, mainly with regard to the immediate neonatal condition. A significant delay (nearly 2 min) from incision to fetal extraction was observed for EPCD compared with TPCD. Although 2 min does not necessarily have clinical significance in general, it can be substantial for fetal extraction and may indicate a difficult extraction. Apgar score at 5 min and mean cord artery pH at birth were similar between the two techniques; still, the higher incidence of pH < 7.2 observed in this study is a source of concern. However, this outcome was reported in two trials only [24,25]. It is not clear why pH < 7.2 was chosen instead of pH < 7.0, which has a much more substantial clinical significance [27]. Nevertheless, the significant difference between the two techniques was reported for only one trial, and there was no difference in pH < 7.2 between the groups in the other trial. PH < 7.0 was apparently not observed in these two trials. Additional neonatal consequences were not reported for all the trials included. This may be of particular concern, particularly when a delay in fetal extraction was recorded. In a number of cases, it was necessary to use forceps or spatulas to assist delivery of the fetal head because of a narrow extraction space. The results for such extraction, and whether it led to any particular trauma, were not reported in these particular trials. It should be pointed out that a narrow fetal extraction space was mostly reported in trials where the rectus muscles were separated laterally on one side only, as compared with separating the paired rectus muscles in the midline and the fascia transversely. In addition, an increased number of cases with cord artery pH < 7.2 were also reported in a trial where the rectus muscles were separated on one side only, lateral to the linea alba [25].

All the included trials investigated short-term outcomes only, and for that reason, we were unable to analyze long-term outcomes regarding EPCD. It is reasonable to assume that avoiding peritoneal opening has long-term advantages. Accordingly, it would be interesting in the future to examine the impact of leaving the peritoneum intact on future fertility, adhesion formation, and other related consequences, such as the occurrence of ectopic pregnancies, including scar pregnancies. Additionally, it is worth examining whether an intact peritoneum affects uterine scar healing and the invasive behavior of the placenta in cases of future placenta accreta sequence.

A recently published meta-analysis of seven RCTs also compared EPCD and TPCD techniques [28]. The researchers reported several outcomes that differed entirely from ours, including time to delivery, change in hemoglobin, and length of hospitalization. They also presented Apgar scores at 1 min, whereas this measure could not be analyzed in our study due to the absence of standard deviation data. Notably, although their review was described as including only RCTs, one retrospective study was incorporated, which may account for some of the discrepancies observed. In contrast to their work, we reported cord pH at birth and placed greater emphasis on neonatal outcomes, which we consider vital when comparing the two techniques.

### 4.3. Strengths and Limitations

The Apgar score at 1 min was planned to assess the neonatal condition at birth; however, this outcome was not meta-analyzed due to insufficient data for calculating the standard deviation. Furthermore, though the current study is the largest to examine EPCD with a high level of evidence, the incidence of several additional operative outcomes is relatively low, such as for cord artery pH < 7.0 (0.3–0.9%) [27] and bladder injury (0.44%) [29]. In order to show a significant difference in these outcomes between the two techniques, large trials with larger numbers of participants are needed. Furthermore, the number of preterm deliveries included in the meta-analysis was small; therefore, data on the use of EPCD in preterm deliveries are limited. Within this context, EPCD may be associated with additional challenges, including smaller uterine size, altered bladder anatomy, and the potential requirement for instrumental assistance, which may elevate the risk of adverse maternal and neonatal outcomes, particularly among neonates delivered preterm. We also acknowledge that ethnicity varied across the included trials, with most studies conducted in Europe and Asia. However, we believe that this heterogeneity is unlikely to have a meaningful impact on the results of the meta-analysis, as outcomes assessed are not expected to differ substantially. Additionally, due to the high heterogeneity among several outcomes, both maternal and neonatal findings should be interpreted with caution. Moreover, because fewer than 10 studies were included, neither formal tests for publication bias nor funnel plots were performed, as their interpretation was unreliable in 7 studies [30]. Finally, the included studies reported short-term findings, while long-term outcomes remain unavailable for both the newborn and the woman, particularly regarding future fertility, trial of labor after cesarean, and abdominal adhesions.

## 5. Conclusions

We think that EPCD deserves to be reconsidered, particularly if separation of the paired rectus muscles is achieved in the midline to avoid possible narrowing of the space from which the fetus is extracted. To achieve this, large RCTs are still required to determine whether this technique is feasible for routine clinical practice, to evaluate its immediate fetal effects, to assess long-term maternal and fetal outcomes, and to examine the cost-effectiveness of the extraperitoneal approach compared with conventional transperitoneal CD. However, such trials should include surgeons with substantial experience in the technique; given the limited number of clinicians well trained in and experienced with EPCD, conducting large future RCTs may be challenging.

## Figures and Tables

**Table 1:** Basic demographic and obstetric variables of the women included in the meta-analysis.

Variables	No. of Studies Analyzed	EPCD Group N-EPCD (%)	TPCD Group N-TPCD (%)	WMD or RR ^a^ (95% CI)	*p* Value	I^2 b^
Maternal age, mean (SD), y	5	30.1 (3.5) (n = 274)	30.9 (3.0) (n = 274)	−1.05 (−1.33, −0.76)	<0.001	0
Maternal weight, mean (SD), kg	2	78.8 (0.8) (n = 108)	80.5 (2.8) (n = 108)	−2.45 (−5.43, 0.52)	0.10	56.2
Body mass index, mean (SD), kg/m^2^	6	28.1 (3.5) (n = 299)	28.8 (4.0) (n = 299)	−0.52 (−1.73, 0.68)	0.39	85.5
Gestational age at delivery, mean (SD), wk	6	37.9 (1.3) (n = 299)	38.0 (1.1) (n = 299)	−0.02 (−0.28, 0.25)	0.91	58.1
Nulliparous, (%)	4	132/246 (53.7)	113/246 (45.9)	0.52 (0.34, 0.70)	<0.001	86.4
Indication of cesarean delivery						
One previous cesarean delivery (%)	2	49/185 (26.5)	63/185 (34.1)	0.26 (0.20, 0.33)	<0.001	0
Two previous cesarean deliveries (%)	2	11/185 (6.0)	20/185 (10.8)	0.55 (0.27, 1.12)	0.10	0
History of any cesarean delivery (%)	5	112/304 (36.8)	142/304 (46.7)	0.37 (0.28, 0.46)	<0.001	60.6
Breech (%)	3	32/190 (16.8)	28/190 (14.7)	1.15 (0.74, 1.79)	0.54	0
Preeclampsia or fetal growth restriction (%)	2	25/132 (18.9)	16/132 (12.1)	1.62 (0.62, 4.18)	0.32	13.0
Others (%)	2	12/132 (9.1)	12/132 (9.1)	0.83 (0.17, 3.96)	0.82	67.6

^a^ Relative risks (RRs) with 95% of confidence intervals (CI) were used as the pooled effect for categorical outcomes, and weighted mean difference (WMD) with 95% of CI was used as the pooled effect for continuous outcomes. ^b^ I^2^ (%) was used to identify heterogeneity (a value > 50% was judged as showing significant heterogeneity between studies). EPCD, extraperitoneal cesarean delivery; TPCD, transperitoneal cesarean delivery.

**Table 2:** Neonatal outcomes.

Outcomes	No. of Studies Analyzed	EPCD Group N-EPCD (%)	TPCD Group N-TPCD (%)	WMD or RR ^a^ (95% CI)	*p*-Value	I^2 b^
Skin incision to delivery time, mean (SD), min	5	7.5 (5.0) (n = 249)	6.2 (3.7) (n = 249)	2.23 (0.40, 4.06)	0.017	98.0
Apgar score at 5, mean (SD), min	5	9.3 (0.6) (n = 252)	9.3 (0.6) (n = 257)	−0.00 (−0.002, 0.002)	0.91	85.7
Umbilical cord artery pH ^c^ (SD)	2	7.302 (0.02) (n = 133)	7.30 (0.00) (n = 141)	0.01 (0.004, 0.016)	0.001	0
Umbilical cord artery pH < 7.2 ^d^ (%)	2	13/164 (7.9)	4/172 (2.3)	3.15 (1.01, 9.76)	0.047	0
Birth weight, mean (SD), g	5	2889.9 (401.6) )n = 252)	2958.3 (295.9) (n = 257(	−74.23 (−206.83, 58.37)	0.27	45.5

^a^ Relative risks (RRs) with 95% of confidence intervals (CIs) were used as the pooled effect for categorical outcomes, and weighted mean difference (WMD) with 95% of CI was used as the pooled effect for continuous outcomes. ^b^ I^2^ (%) was used to identify heterogeneity (a value > 50% was judged as showing significant heterogeneity between studies). ^c^ The entire database on umbilical cord artery pH included 274 neonates that were born to 264 women. ^d^ The entire database on umbilical cord artery pH < 7.2 included 336 neonates that were born to 326 women. EPCD, extraperitoneal cesarean delivery; TPCD, transperitoneal cesarean delivery.

**Table 3:** Secondary outcomes.

Outcome	No. of Studies	Totals EPCD Group vs. TPCD Group	WMD or RR ^a^ (95% CI)	*p* Value	I^2 b^
Operative time, mean (SD), min	5	38.5 (18.3) vs. 36.6 (13.0) (n = 249 vs. n = 249)	2.56 (−3.39, 8.51)	0.40	93.4
Intraoperative nausea/vomiting, N- EPCD/N- TPCD (%)	2	4/165 (2.4) vs. 31/155 (20.0) (n = 165 vs. n = 155)	0.13 (0.01, 2.92)	0.28	76.8
Estimated blood loss, mean (SD), mL	2	696.2 (1.9) vs. 672.8 (57.2) (n = 92 vs. n = 92)	29.11 (−61.69, 119.91)	0.53	36.6
VAS score 6 h after surgery, mean (SD)	3	3.9 (2.3) vs. 6.7 (1.2) (n = 109 vs. n = 109)	−2.61 (−5.53, 0.31)	0.079	99.6
VAS score 12 h after surgery, mean (SD)	4	2.7 (1.1) vs. 4.1 (0.2) (n = 134 vs. n = 133)	−1.29 (−2.52, −0.07)	0.039	98.1
VAS score 18 h after surgery, mean (SD)	3	2.0 (0.3) vs. 3.2 (0.2) (n = 109 vs. n = 109)	−1.24 (−1.82, −0.65)	0.001	81.5
VAS score 24 h after surgery, mean (SD)	4	2.7 (0.9) vs. 4.4 (1.2) (n = 205 vs. n = 206)	−1.56 (−2.12, −1.01)	<0.001	78.99
Time until gas passage, mean (SD), h	3	7.4 (2.7) vs. 14.7 (2.7) (n = 164 vs. n = 164)	−4.64 (−5.53, −3.76)	<0.001	33.03
Change in hemoglobin, mean (SD), g\dL	5	1.2 (0.7) vs. 1.2 (0.5) (n = 249 vs. n = 249)	−0.04 (−0.32, 0.24)	0.79	70.7
Length of hospital stay, mean (SD), day	4	3.5 (1.8) vs. 3.3 (1.6) (n = 224 vs. n = 224)	0.28 (−0.52, 1.08)	0.49	90.5

^a^ Relative risks (RRs) with 95% of confidence intervals (CIs) were used as the pooled effect for categorical outcomes, and weighted mean difference (WMD) with 95% of CI was used as the pooled effect for continuous outcomes. ^b^ I^2^ (%) was used to identify heterogeneity (a value > 50% was judged as showing significant heterogeneity between studies). VAS, Visual Analogue Scale to measure pain intensity. EPCD, extraperitoneal cesarean delivery; TPCD, transperitoneal cesarean delivery.

## Data Availability

No new data were created or analyzed in this study.

## Supplementary Materials

The following supporting information can be downloaded at: https://www.mdpi.com/article/10.3390/jcm15010191/s1, Figure S1: Risk of bias summary and graph. Table S1: Baseline descriptive data of the enrolled trials. Checklist S1: PRISMA checklist.
